# Obesity Phenotyping in Children and Adolescents: Next Steps Towards Precision Medicine in Pediatric Obesity

**DOI:** 10.3390/nu18020303

**Published:** 2026-01-18

**Authors:** Leslie Saba, Andres J. Acosta, Aaron S. Kelly, Seema Kumar

**Affiliations:** 1 Division of Pediatric Endocrinology, Department of Pediatric and Adolescent Medicine, Mayo Clinic, Rochester, MN 55905, USA; 2 Precision Medicine for Obesity Program, Division of Gastroenterology and Hepatology, Department of Medicine, Mayo Clinic, Rochester, MN 55905, USA; 3 Center for Pediatric Obesity Medicine, Department of Pediatrics, University of Minnesota, Minneapolis, MN 55454, USA

**Keywords:** pediatric obesity, treatment response heterogeneity, obesity phenotypes, precision medicine

## Abstract

Pediatric obesity is an increasingly prevalent, chronic, and multifactorial disease. Achieving successful and sustained weight reduction with current interventions remains challenging due to significant heterogeneity in treatment response. This review summarizes current evidence describing variability in outcomes across lifestyle, pharmacologic, and metabolic/bariatric surgery interventions in children and adolescents, and examines key biological, metabolic, behavioral, environmental, and psychosocial factors that influence response. In adults, recent findings on energy balance obesity phenotypes (characterized by abnormal satiation, abnormal postprandial satiety, abnormal hedonic eating, and reduced energy expenditure) have demonstrated promise in predicting weight loss outcomes and guiding tailored interventions. However, data on obesity phenotyping within children and adolescents remain limited. Addressing this gap is essential for advancing precision medicine approaches in pediatric obesity, with the potential to improve treatment selection, enhance effectiveness, and optimize long-term clinical outcomes.

## 1. Introduction

Pediatric obesity is a chronic, multifactorial disease with a rising global prevalence, increasing from 2% in 1990 to 6.8% in 2021 in children aged 5 to 14 years and representing a more than tripling of rates over this period [1]. Globally, approximately 1 in 5 children and adolescents are affected by overweight or obesity, with prevalence varying across countries and higher rates observed in high-income countries and in those with a very high Human Development Index [2]. Beyond its association with short-term comorbidities, such as type 2 diabetes, metabolic dysfunction-associated steatotic liver disease (MASLD), dyslipidemia, hypertension, obstructive sleep apnea, and mental health disorders, pediatric obesity confers an increased risk of future cardiovascular disease, cardiovascular mortality, and all-cause mortality in adulthood [3,4,5].

Despite increasing prevalence of pediatric obesity, achieving successful and sustained weight loss outcomes with current interventions is challenging. Lifestyle interventions in children with obesity frequently result in modest and heterogenous weight loss [6]. This limited response reflects powerful biological adaptations to energy restriction that favor weight regain, and exposure to highly obesogenic environments that overwhelm individual behavioral change [7]. High attrition rates in pediatric weight management programs further attenuate observed efficacy by limiting treatment dose, continuity, and ability to sustain behavioral change over time [8]. Additionally, there are variable responses to pharmacologic therapy and metabolic and bariatric surgery (MBS) [6]. This heterogeneity in treatment response underscores the limitations of a “one-size-fits-all” approach and highlights the need for a more personalized framework for obesity care in the pediatric population [9,10]. By characterizing obesity phenotypes and identifying factors that influence treatment response, precision medicine can be used to tailor treatment strategies to an individual’s biological, behavioral, and environmental profile.

This narrative review summarizes current evidence on the heterogeneity of response to pediatric obesity interventions, identifies key factors contributing to this variability, and explores how obesity phenotypes may inform individualized care.

## 2. Methods

A comprehensive literature search was performed to identify relevant studies on pediatric obesity phenotyping and treatment response. The search strategy included the use of two major biomedical databases: PubMed and Web of Science. These databases were selected for their broad coverage of clinical, translational, and epidemiological research. Searches were conducted using combinations of the following main keywords and controlled vocabulary: “pediatric obesity,” “obesity phenotypes,” “precision medicine,” “treatment response heterogeneity,” “lifestyle intervention,” “pharmacologic therapy,” and “bariatric surgery.” Reference lists of included articles and relevant reviews were also screened to identify additional studies. Only articles published in English were reviewed. No restrictions were placed on publication date or study design to ensure a thorough synthesis of the available evidence.

## 3. Drivers of Pediatric Obesity

The increasing prevalence of pediatric obesity is driven by complex, interacting factors at the individual, family, community, and policy levels [11]. Shifts in dietary patterns with increased consumption of ultra-processed foods, sugar-sweetened beverages, and energy-dense, low-fiber, high-fat diets are associated with an increased risk of obesity in children and adolescents [12,13,14]. Decreased physical activity and increased screen time further contribute to the risk of overweight or obesity [15,16]. Variants in genes involved in energy homeostasis and appetite regulation, primarily affecting the leptin-melanocortin pathway, are associated with an increased risk of obesity in children [17]. Dysregulation of hormonal pathways such as leptin, ghrelin, insulin, peptide YY, and glucagon-like peptide-1 (GLP-1) can also affect appetite and satiety [18]. Additionally, psychosocial factors including family dietary patterns, socioeconomic status, and food insecurity can further influence eating behaviors and physical activity levels [11].

Increased total energy intake, coupled with reduced physical activity, creates a positive energy balance which promotes excess fat accumulation and results in an increased risk of obesity [19]. Excess adipose tissue in children with obesity contributes to increased inflammation and cardiometabolic disease through adipocyte hypertrophy and dysfunction, macrophage infiltration, and secretion of pro-inflammatory cytokines, adipokines, and inflammatory markers such as IL-6, TNF α, leptin, and high-sensitivity C-reactive protein [20,21]. This inflammatory state can occur early in childhood obesity, resulting in impaired insulin signaling and promoting the development of insulin resistance [21].

## 4. Heterogeneity in Treatment Response

Evidence from large longitudinal cohorts highlights the variability in response to lifestyle interventions, even within structured, standardized programs. In a registry-based study of over 12,000 children and adolescents with overweight or obesity (median age 11.5 years; median BMI z-score 2.06) receiving care from 148 centers specializing in obesity from Germany, Austria, or Switzerland, response after two years of outpatient lifestyle intervention varied significantly with 36% not achieving a reduction in BMI z-score, 45% achieving a modest average decrease of −0.23, and 19% demonstrating a more pronounced average reduction of −0.61 [22]. The intensity of lifestyle intervention (often defined as hours of contact) has been found to influence outcomes, with high-intensity lifestyle programs yielding the greatest average BMI reductions. The American Academy of Pediatrics therefore recommends a minimum of 26 h of intensive health behavior and lifestyle treatment over a 3–12-month period [11]. However, even in those receiving at least 52 h of contact, variability persisted, with mean BMI z-score reductions ranging from −0.05 to −0.34, as reported in a systematic review of five studies involving a total of 563 participants who received approximately 67–114 contact hours over 12 months [23].

Response to pharmacologic therapy further illustrates this heterogeneity. GLP-1 receptor agonists, including semaglutide and liraglutide, are currently approved for management of obesity in adolescents aged 12 years and older, and have demonstrated significant mean efficacy, yet with wide interindividual differences. In the STEP TEENS trial, weekly semaglutide 2.4 mg led to a mean BMI reduction of −16.1% at 68 weeks. While 76% of participants achieved at least a 5% reduction and 40% achieved at least a 20% reduction in BMI, almost a quarter experienced <5% reduction in BMI with semaglutide [24]. Modest weight regain was noted after 7 weeks post-semaglutide discontinuation (mean BMI reduction from baseline of 13.2% at week 75 versus the 16.1% reduction at week 68) [24]. Similar variability in BMI response is noted from the randomized trial involving liraglutide, where 43.3% of adolescents achieved ≥5% BMI reduction, compared to 26.1% who achieved ≥10% reduction in BMI from baseline after 56 weeks of treatment with liraglutide 3 mg [25]. Greater increase in BMI z-score was noted in patients who discontinued liraglutide than those who discontinued placebo during the 26 week follow-up period after the 56 week treatment period (0.22 versus 0.07; estimated treatment difference, 0.15; 95% CCI, 0.07–0.23) [25]. The randomized trial evaluating phentermine/topiramate showed comparable heterogeneity, with 47%, 43%, and 28% of participants achieving ≥5%, ≥10%, and ≥15% BMI reductions, respectively, at the highest dose of 15/92 mg [26].

MBS outcomes in adolescents also highlight that individual BMI trajectories differ widely over long-term follow-up. In a study evaluating outcomes following Roux-en-Y gastric bypass in adolescents ages 13 to 19 years using the Teen-Longitudinal Assessment of Bariatric Surgery (Teen-LABS) multicenter cohort, approximately 60% of adolescents maintained ≥20% weight reduction at 5 years, whereas 4% experienced <5% reduction, and another 4% exhibited weight regain over the same period [27]. Another study evaluating long-term 10-year outcomes following MBS (gastric bypass and sleeve gastrectomy) using the Teen-LABS cohort identified four distinct BMI trajectories over time using latent class analysis, with one group demonstrating increased BMI at 10 years compared to baseline whereas another group had BMI approximately 40% lower than at baseline [28]. Adolescents with severe obesity demonstrated marked inter-individual variation in BMI change in response to lifestyle (range: −25.4% to 5.0%), pharmacotherapy (range: −10.8% to 12.9%), and metabolic and bariatric surgery (range: −50.2% to −13.3%) [6].

## 5. Factors That Influence Heterogeneity in Treatment Response

**Figure 1:**
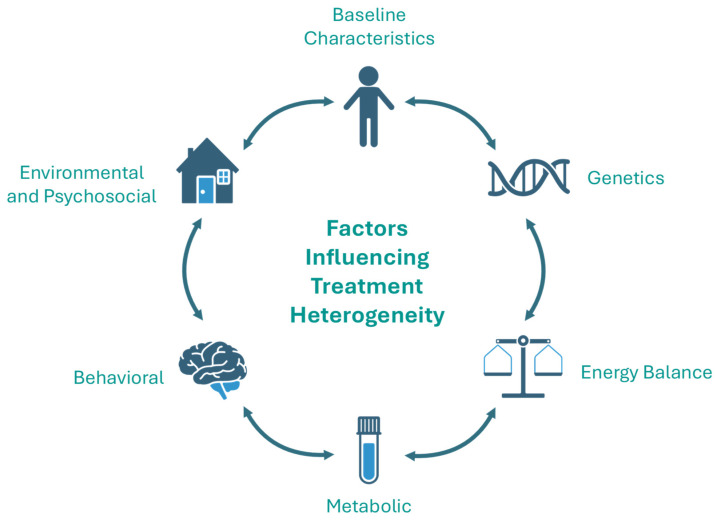
Key Factors Influencing Treatment Heterogeneity.

Obesity is a multifactorial disease that reflects the interplay of genetic, behavioral, environmental, and psychosocial determinants which can collectively influence an individual’s weight and metabolic risk (Figure 1). Each of these factors may contribute to the variability in response to interventions in children and adolescents with obesity (Table 1).

### 5.1. Baseline Characteristics

Certain patient characteristics have been found to affect response to treatment. Younger children, particularly those under 12 years at initiation of therapy, have demonstrated greater BMI reduction in response to lifestyle interventions compared to adolescents [22,29,30]. Decreased parental involvement and increased autonomy in adolescents compared to younger children, as well as increased fat mass in females with pubertal development may account for these differences [52,53]. Research on sex differences in response to lifestyle interventions has produced mixed results, showing increased or decreased weight loss in males, or no difference between sexes [22,31,54]. One study reported that children and adolescents with lower baseline BMI experienced greater reductions in BMI following lifestyle intervention, while another study found that higher baseline BMI was associated with a more favorable response [22,31]. Greater initial reduction in BMI z-score (>5%) within the first 3 months of lifestyle intervention was associated with higher chances of BMI reduction at 2 years [22].

In a post hoc analysis, baseline characteristics such as age, sex, race, ethnicity, pubertal stage, or stage of obesity, did not have a predictive impact on response to liraglutide after 56 weeks of therapy in adolescents with obesity [45]. Early responders to liraglutide (defined as ≥4% reduction in BMI at week 16) were more likely to achieve greater BMI and weight reductions at week 56 compared to early non-responders [45]. Similarly, a secondary analysis of a randomized, placebo-controlled trial of the response to phentermine/topiramate in adolescents with obesity did not find that baseline characteristics were predictive of BMI response after 56 weeks [55]. On the other hand, female sex was found to be a favorable predictive factor of BMI reduction in adolescents with severe obesity receiving exenatide for 3 months, while other characteristics such as age, baseline BMI, and BMI percent change at 1 month did not have a significant predictive value [46]. With regard to MBS, a recent prospective observational cohort study found that a higher preoperative BMI was associated with reduced odds (OR 0.89, 95% CI 0.79–0.97, *p* = 0.03) of achieving >35% reduction in BMI z-score at 12 months after surgery (87.7% laparoscopic sleeve gastrectomy, 12.3% Roux-en-Y gastric bypass) [48].

### 5.2. Genetics

The role of genetic variants in modulating response to obesity interventions is an area of growing research interest. These genetic factors have been classically categorized into three groups: monogenic obesity, syndromic obesity, and polygenic obesity. Monogenic obesity refers to single-gene variants that commonly affect the leptin-melanocortin pathway, for example in MC4R, LEPR, POMC, or PCSK1 genes, that disrupt key regulators of appetite and energy homeostasis [17]. They are inherited in a Mendelian inheritance pattern and account for approximately 5% of obesity cases, though the prevalence has been reported to reach up to 30% in certain populations [56,57,58]. These cases typically present in early childhood before 5 years of age with severe, rapid-onset obesity and hyperphagia [59]. Syndromic forms of obesity, while also inherited in a Mendelian pattern, are typically associated with characteristic signs and symptoms affecting multiple systems, such as in Prader–Willi syndrome and Bardet–Biedl syndrome (BBS) [60]. With regard to response to obesity interventions, a cohort of 9 children with MC4R variants had a similar degree of weight loss compared to 46 age- and gender-matched children without MC4R variants following a 1-year lifestyle intervention (mean BMI z-score decrease of 0.3 versus 0.4, respectively; *p* = 0.318 based on intention-to-treat analysis); however, the weight loss was not sustained post-intervention in children with MC4R variants [61]. Pharmacologic interventions that target these affected energy balance pathways highlight the potential benefits of precision medicine approaches to obesity care. For example, setmelanotide, an MC4R agonist, is approved for individuals with BBS and monogenic obesity due to POMC, LEPR, or PCSK1 deficiency, with studies involving pediatric participants showing 80% of those with POMC-deficiency, 45% with LEPR deficiency, and 32.3% of BBS participants achieving at least 10% reduction in body weight after 1 year of treatment [62,63]. Children and adolescents with syndromic and monogenic obesity have also demonstrated significant BMI reductions following bariatric surgery, but these data are derived from very small studies and long-term weight outcomes are not available [64,65,66].

In contrast to monogenic and syndromic forms of obesity, polygenic obesity results from the cumulative effects of multiple gene variants which interact with epigenetic and environmental factors and contribute to an increased risk of obesity [32]. Polygenic obesity is not inherited in a Mendelian pattern and is instead inherited in a similar manner to other complex diseases and traits [17]. It is the most common form of obesity and is further distinguished from monogenic and syndromic forms by the lack of distinctive clinical features [67]. However, emerging evidence suggests that there is heterogeneity even within monogenic cases of obesity, and that there are likely polygenic influences that affect clinical presentations [17]. Results from genome-wide association studies (GWAS) of BMI in over 5 million individuals identified a multi-ancestry polygenic score that explained 17.6% of BMI variation in a European-ancestry cohort, with explained variance ranging from 2.2% to 16% in other populations [68]. Another polygenic risk score for obesity, developed by Khera et al., showed that adults in the top 10% of the score had a 25-fold greater risk of severe obesity than those in the bottom decile [69]. Additionally, in a longitudinal cohort following children from birth to 18 years, stratifying individuals into top, middle, and bottom deciles of the score revealed distinct weight trajectories that emerged in early childhood and reached a difference of 12 kg by age 18 [69]. These polygenic variants often affect appetite regulation, energy expenditure, and eating behaviors, and their overall effects can be further influenced by lifestyle and environmental factors such as diet, sleep, exercise, and socioeconomic status [70]. For example, a study performed in children ages 4–5 years showed that children with high-risk FTO alleles, one of the earlier and more robustly BMI-associated gene loci, had 25% higher food intake compared to those with the low-risk FTO allele [17,71]. A growing area of research is evaluating the influence of these polygenic obesity loci and the differential responses to lifestyle, pharmacologic, and surgical therapies. One systematic review evaluating the influence of 92 polymorphisms in children and adolescents with overweight or obesity identified 24 genetic loci that influenced BMI or body composition response to lifestyle intervention [32]; however, other studies have demonstrated little to no ability of polygenic variants to predict responses to lifestyle intervention in children [72,73,74]. Another study found that carriers of the FTO obesity-predisposing allele were less likely to have an increase in BMI z-score following lifestyle intervention compared with non-carriers [31]. From a pharmacologic standpoint, one study did not show utility of polygenic risk scores in predicting response to metformin in children with obesity [75]. There are no data on the role of polygenic risk scores in predicting response to other obesity medications or to MBS in the pediatric population. In adults, polygenic scores for BMI and type 2 diabetes were not associated with weight loss response to GLP-1 receptor agonists [76]. The same study found that a higher BMI polygenic score was associated with slightly less weight loss after MBS, though the effect was small (approximately 0.7% less weight lost per standard deviation of the score) [76]. Integrating polygenic risk scores with other phenotypic markers may help stratify which children may respond to a particular treatment approach [77].

### 5.3. Energy Balance

Energy balance, defined as the relationship between energy intake and energy expenditure, is another factor that can influence weight loss response [78]. It is well-established that increased intake of energy-dense foods and increased sedentary behaviors can contribute to increased obesity risk [15]. In line with these risk factors, increased baseline intake of soft drinks was found to negatively predict weight loss in response to a family-based behavioral lifestyle and dietary intervention program, whereas higher baseline physical activity and greater daily water intake were associated with greater weight loss [33]. There is variation in dietary recommendations for pediatric obesity and can include caloric restriction, the traffic light diet (categorizing foods into green, yellow, or red based on energy density), and more intensive approaches such as very low-energy diets [79]. A recent systematic review evaluating dietary energy content recommendations in pediatric weight management interventions found that dietary interventions with greater energy deficits were associated with greater reductions in BMI, while interventions centered on general nutrition education without a defined energy target resulted in a slight increase in BMI [34]. Energy-targeted approaches such as hypocaloric, basal-metabolic-rate-based, and normocaloric dietary interventions achieved clinically significant average BMI reductions of at least 5% [34]. Furthermore, diet quality has been shown to be a significant predictor of response to lifestyle intervention in adolescents with overweight and obesity. In one study involving 117 adolescents receiving a multidisciplinary intervention consisting of diet, physical activity, and psychosocial support, changes in diet quality index explained 98.1% of BMI z-score changes and 95.1% of fat mass index changes after 13 months [35]. In a study of 72 adolescents participating in a 16-week behavioral weight loss trial, higher initial frequency of intake of vegetables and increased frequency of intake of fruits and reduced-calorie snack foods over the first 4 weeks of treatment accounted for 43% of the variance in BMI reduction at 12 weeks (*p* < 0.001) [36]. With regard to energy expenditure, increased physical activity is associated with greater reduction in percent body fat, but not BMI, in children and adolescents with overweight and obesity [80]. Additionally, higher baseline cardiorespiratory fitness is associated with greater reduction in BMI and fat mass in adolescents with obesity receiving multidisciplinary weight management intervention involving physical activity, nutritional recommendations, and psychological support [37]. In adolescents and young adults (aged 12–21 years) undergoing sleeve gastrectomy, self-reported pre-operative exercise of at least 5 h per week predicted greater weight loss at 6 months and marginally at 12 months postoperatively [49].

Obesity pharmacotherapies primarily drive weight loss through a decrease in energy intake. However, pediatric studies rarely include objective measures of energy intake or expenditure and the ability to determine how differences in energy balance behaviors contribute to the variability in treatment response is uncertain. Identifying and validating energy balance phenotypes, such as “low-intake” and “high-expenditure” phenotypes, in children may provide insight into who is more likely to respond to a particular intervention and could guide future personalized approaches.

### 5.4. Metabolic

There is significant heterogeneity in metabolic health among children and adolescents with obesity, with a subset demonstrating a favorable cardiometabolic profile, commonly referred to as metabolically healthy obesity (MHO), while others exhibit metabolically unhealthy obesity (MUO), characterized by one or more metabolic abnormalities such as dyslipidemia, hypertension, impaired glucose regulation, or insulin resistance [81,82,83]. Children with the MHO phenotype tend to be younger, prepubertal, and have lower BMI, waist circumference, and body fat measurements compared to those with MUO [84,85]. These metabolic differences may play a role in the variability observed in weight loss and metabolic response to obesity interventions.

In a retrospective study of 733 children and adolescents with overweight and obesity without diabetes, the presence of baseline prediabetes predicted greater improvement in BMI relative to the 95th percentile over 12 months compared with peers without prediabetes enrolled in a weight management program [38]. Conversely, a retrospective study of 134 adolescents with obesity demonstrated that higher baseline fasting insulin and homeostasis model assessment insulin resistance (HOMA-IR) were independently associated with poorer response to lifestyle therapy, with each 10-unit increase in fasting insulin and 1-unit increase in HOMA-IR conferring 3.13-fold and 1.64-fold greater odds of nonresponse (defined as an increase in BMI z-score), respectively [39]. Consistent with these findings, another study reported that insulin resistance and other components of the metabolic syndrome, including increased waist circumference, elevated blood pressure, and hypertriglyceridemia, were significant negative predictors of weight loss among children with obesity participating in lifestyle interventions [40]. Additionally, baseline leptin levels have been inversely associated with weight loss response to lifestyle intervention in children with obesity [41].

Post hoc analysis of the adolescent liraglutide trial demonstrated that the likelihood of achieving ≥5% or ≥10% BMI reduction with liraglutide compared to placebo was lower among participants with baseline hyperglycemia, however, these results were not statistically significant [45]. Similarly, a systematic review and meta-analysis reported that GLP-1 receptor agonists were more likely to result in greater body weight reduction in children with obesity compared to those with type 2 diabetes [86]. These findings are limited by the small number of available studies, the relatively fewer participants with hyperglycemia or type 2 diabetes, and the lower dose of liraglutide used for treatment of type 2 diabetes than for obesity treatment. Glycemic status was not identified as a significant predictor of BMI reduction in adolescents with obesity treated with phentermine/topiramate [55]. Lower baseline leptin response to meals has been associated with greater weight loss maintenance in those treated with exenatide [47].

In adolescents undergoing laparoscopic sleeve gastrectomy, higher baseline systolic blood pressure predicted greater weight loss by change in absolute BMI and BMI z-score at both 6 and 12 months, while elevated hemoglobin A1c was associated with greater reduction in BMI z-score at 6 months [50]. However, other metabolic parameters, such as fasting glucose, liver function, triglycerides, and waist circumference, were not associated with a predictive outcome [50]. Another study examining predictors of achieving at least a 25% relative reduction in BMI z-score at 12 months following MBS found that preoperative lipid profiles, HbA1c, and liver enzyme levels were not significant determinants of postoperative weight loss [48].

### 5.5. Eating Behavior

Eating behavior phenotypes may also influence weight loss response in children and adolescents with obesity. Eating behaviors develop rapidly from infancy to school age and can be affected by biological, psychosocial, and developmental factors [87]. A prospective cohort study of eating behaviors among children showed that children with a higher BMI at age 4 years were more likely to have greater food responsiveness, greater food enjoyment, and decreased satiety responsiveness at the age of 10 years [88]. Additionally, emotional eating was identified as both a predictor and a consequence of increased BMI in this cohort [88].

A recent study identified three distinct eating behavior profiles among children and adolescents with overweight or obesity enrolled in a 10-week lifestyle intervention camp: low, medium, and high food approach, based on their degree of food responsiveness and emotional eating [89]. Those in the high food approach group were more likely to be younger, reported lower baseline quality of life, and had the highest BMI standard deviation scores [89]. While this group did not achieve greater BMI reduction than other profiles in response to the lifestyle intervention, they experienced the largest improvements in quality of life and overeating behaviors [89]. These findings highlight the potential value of tailoring interventions to individual eating behavior phenotypes.

Currently approved obesity pharmacotherapies target physiologic pathways involved in regulating appetite and satiety, for example, phentermine/topiramate acts centrally, while GLP-1 receptor agonists have both central and peripheral effects through the hypothalamus and gastrointestinal tract [90,91]. In pooled data from two clinical trials of exenatide in adolescents with severe obesity, greater baseline appetite was associated with a more pronounced reduction in BMI after 3 months of therapy, whereas baseline satiety levels were not predictive of treatment response [46]. On the other hand, currently published studies on adolescents undergoing MBS do not suggest that changes in appetite at baseline or eating behaviors postoperatively correlate with BMI reduction or maintenance after surgery [92,93].

### 5.6. Environmental and Psychosocial Factors

Environmental and psychosocial factors are important considerations in children and adolescents with obesity. Socioeconomic status, the community and home environments, and family dynamics collectively influence both access to and engagement with interventions [11]. Lower socioeconomic status and housing insecurity have been associated with higher dropout rates and nonadherence to pediatric weight management interventions [94,95,96]. One study showed that children in households with food insecurity had an increase in their BMI by 0.5 kg/m^2^, or 2.1 %BMIp_95_, per year compared to food secure households following a multidisciplinary lifestyle intervention [42]. Moreover, children and adolescents with less social deprivation were more likely to have greater reduction in BMI z-score following participating in outpatient lifestyle intervention [22]. In adolescents who underwent laparoscopic adjustable gastric banding, the presence of family conflict was associated with reduced postoperative weight loss [51].

Parental characteristics, such as parental obesity and education level, have also been associated with response or adherence to weight management programs [43,97,98]. In a retrospective study, the presence of obesity-related comorbidities in both parents was associated with a 12.6-fold higher likelihood of nonresponse to lifestyle interventions among children with obesity [39]. These findings likely reflect both shared genetic susceptibility and environmental factors, such as the home food environment, parental modeling of health behaviors, and psychosocial stress, that may together shape treatment response [11]. Greater parental involvement in pediatric weight management programs has been associated with more pronounced weight loss compared to less parental participation, though evidence in adolescents remains limited and warrants further investigation [44,99]. Accordingly, current pediatric clinical practice guidelines emphasize family-based behavioral interventions as a central component of obesity treatment in children and adolescents [11].

## 6. Obesity Phenotyping

The interaction between these intrinsic and extrinsic factors gives rise to distinct obesity phenotypes that vary in severity, comorbidity profile, and treatment responsiveness, and highlight the need to consider these factors collectively when developing treatment strategies for children and adolescents with obesity. At present, obesity phenotyping in this population is typically reliant on anthropometric measures using BMI percentiles for age and sex into class 1 obesity (BMI ≥ 95th percentile to <120% of 95th percentile), class 2 obesity (BMI ≥ 120% to <140% of 95th percentile or BMI ≥ 35 to <40 kg/m^2^, whichever is lower), and class 3 obesity (BMI ≥ 140% of 95th percentile or BMI ≥ 40 kg/m^2^, whichever is lower) [11]. This is often complemented by metabolic markers and classification into metabolically healthy versus unhealthy obesity [81]. However, given the unique combination of biological, behavioral, and environmental influences affecting each child, obesity management requires a personalized approach tailored to the individual’s specific phenotype to achieve the most effective and sustained therapeutic outcomes.

**Figure 2:**
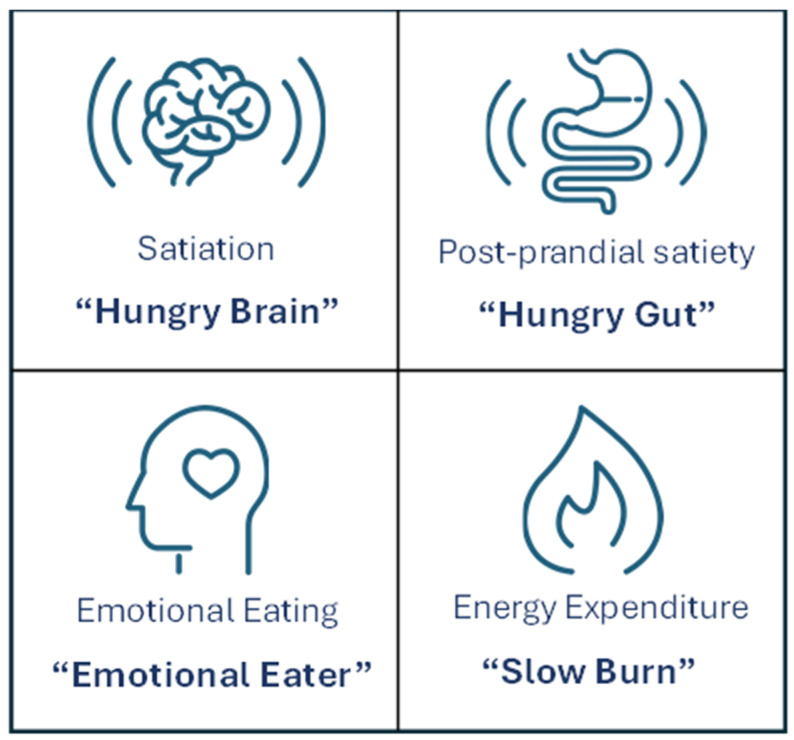
Energy Balance Obesity Phenotypes.

In the adult population, obesity phenotyping studies have identified four subgroups with distinct physiologic and behavioral drivers of obesity: abnormal satiation (hungry brain), abnormal postprandial satiety (hungry gut), abnormal hedonic eating (emotional eating), and abnormal (reduced) energy expenditure [100,101] (Figure 2). These energy balance phenotypes were derived from deep phenotyping participants, which involves 8 h of testing including measurement of resting energy expenditure, body composition using dual-energy X-ray absorptiometry imaging, blood sample collection of fasting and postprandial hormones, genotyping, gastric emptying scans, ad libitum meal, assessment of appetite sensations, and completion of mood and eating behavior questionnaires [102]. Satiation refers to the sensation of fullness that develops during a meal and determines meal size and meal termination; individuals with abnormal satiation require greater caloric intake at each meal before feeling full [102,103]. Satiety describes the process arising after a meal that prevents the return of hunger and affects the timing of the next meal, and those with abnormal postprandial satiety experience an earlier return of hunger and increased frequency of eating in between meals [102,103].

These obesity phenotypes have been shown to predict weight loss response in studies in adults using lifestyle intervention [104], obesity medications [100,105,106], and bariatric endoscopic devices [107,108,109,110]. In a proof-of-concept study, adults with obesity who received phenotype-tailored lifestyle interventions (time-restricted volumetric low-calorie diet for abnormal satiation; low-calorie diet with pre-meal protein supplementation for abnormal postprandial satiety; low-calorie diet with intensive behavioral therapy for abnormal hedonic eating; and low-calorie diet, post-workout protein supplementation, and high-intensity interval training for abnormal resting energy expenditure) had greater weight loss (−3.1 kg [95%CI, −5.1 to −1.1]; *p* = 0.004) compared to those receiving standard lifestyle interventions (low-calorie diet, moderate physical activity, weekly behavioral therapy) [104]. The use of obesity medications guided by an individual’s obesity phenotype (phentermine/topiramate for abnormal satiation, liraglutide for abnormal satiety, naltrexone/bupropion sustained release for abnormal hedonic eating, and phentermine for low energy expenditure) resulted in 1.75-fold greater weight loss at 12 months compared to non-phenotype guided use of these medications [101].

Furthermore, a recent study characterized calories to satiation (CTS) using the ad libitum meal test and validated a machine-learning-assisted gene risk score (GRS) to predict CTS among adults with obesity [102]. Participants completed an ad libitum meal test to quantify calories consumed until satiation. Sex-stratified CTS distributions were divided into quartiles, and “high CTS” was defined as greater than the 75th percentile [102]. Using supervised machine-learning, a model was developed to predict high CTS by integrating weighted genetic variants across 10 genes known to influence appetite and satiation (SIM1, PCSK1, SH2B1, LEPR, UCP2, FTO, TCF7L2, GLP1R, TNFRSF11A, and ADRA2A) [102]. CTS_GRS_, derived from a blood or saliva sample, provides a more accessible alternative to the time-intensive laboratory deep phenotyping protocol. Its clinical utility was evaluated in two previously completed randomized clinical trials and found that high CTS or CTS_GRS_ predicted greater weight loss with phentermine/topiramate after 52 weeks, while those with low CTS or CTS_GRS_ were more likely to respond better to liraglutide at 16 weeks [102]. These advances in adult obesity phenotyping highlight the feasibility and clinical value of a precision-medicine guided treatment approach.

Despite progress in adults, obesity phenotyping across energy balance domains in the pediatric population remains understudied. In adult studies, validated methodologies such as standardized ad libitum buffet meals, behavioral and appetite questionnaires, gastric emptying studies, and measurement of resting energy expenditure, are used to characterize obesity phenotypes [102]. Similar tools evaluating energy intake and expenditure have been applied in pediatrics. For example, a study evaluating the reproducibility of an in-laboratory ad libitum buffet meal in adolescents with obesity found that total energy intake was highly consistent across 3 identical experimental sessions (intraclass correlations of 0.99) [111]. Another study found that ad libitum intake measured during a lab-based palatable buffet meal in children aged 4–6 years was positively associated with body fat assessed by dual-energy X-ray absorptiometry [112]. Structured assessments of appetitive traits, such as food and satiety responsiveness and emotional eating, have also been used to derive behavioral phenotypes in children [113]. Children and adolescents with obesity were found to be more likely to self-report greater hunger and faster eating compared to normal-weight children [114]. Additionally, in a cross-sectional study of children aged 5–12 years with obesity, parent-reported measures of appetitive traits (using the Child Eating Behavior Questionnaire) and psychological/behavioral symptoms (using the Vanderbilt ADHD Scale and Pediatric Symptom Checklist) identified 4 distinct phenotypes: Hedonic Impulsive, Inattentive Impulsive, Hedonic Emotional, and Picky Eating [115]. Similarly, another study identified 3 overeating phenotypes in children with overweight and obesity using self- and parent-reported questionnaires, interview assessments, and a laboratory paradigm assessing eating in the absence of hunger [116]. Regarding energy expenditure, indirect calorimetry remains the gold standard for measuring resting energy expenditure in children and adolescents with obesity [117,118]. Recent studies have shown wide inter-individual variability in resting energy, with most children classified as normometabolic (60.6%), 25.5% as hypermetabolic, and 13.9% as hypometabolic, and evidence suggests that these differences may be influenced by underlying etiologies of obesity [119,120]. These studies demonstrate that energy balance phenotyping is feasible and informative in children. However, pediatric studies remain limited in scope and rarely integrate multidimensional measures of energy balance, including hormonal, behavioral, metabolic, and genetic factors. Future research should aim to standardize and expand these tools to better characterize pediatric obesity phenotypes, establish normative reference data, and ultimately evaluate whether targeting energy balance phenotypes can improve treatment outcomes in children and adolescents with obesity.

It is important to note that most of the referenced studies informing the factors influencing response to obesity intervention and obesity phenotyping were conducted in the United States and Europe, with limited representation from other sociocultural contexts, which may limit the generalizability of these findings.

## 7. Conclusions and Future Directions

There is considerable heterogeneity in treatment response among children and adolescents with obesity, influenced by a complex interplay of genetic, metabolic, behavioral, environmental, and psychosocial determinants. Defining and validating energy-balance obesity phenotypes and replicating adult-phenotype guided treatment approaches through randomized controlled trials in children could enable more precise, individualized treatment strategies. These advances have the potential to improve intervention efficacy and move pediatric obesity care toward a precision medicine framework.

## Figures and Tables

**Table 1:** Summary of Literature on Predictors of Weight Loss Response to Obesity Interventions in Children and Adolescents.

Intervention	Authors, Year	Study Design and Population	Key Predicting Factors	Reference
Lifestyle	Prinz et al., 2023	Registry cohort of 12,453 children and adolescents with overweight/obesity (median age 11.5 years, BMI z-score 2.06, 52.6% girls) who participated in outpatient lifestyle (dietary, physical activity, and behavioral) intervention for up to 2 years	Younger age, lower baseline BMI z-score, larger initial reduction in BMI z-score, less social deprivation predicted moderate/pronounced BMI z-score reduction	[22]
Lifestyle	van de Pas et al., 2022	Longitudinal study evaluating outcomes at 1 and 2 years of multidisciplinary lifestyle (dietary, physical activity, and behavioral) intervention in 83 children (mean age 8.3 years, BMI z-score 4.07, 47% female) and 77 adolescents (mean age 15.2 years, BMI z-score 3.96, 61% female) with severe obesity	Younger age predicted greater reduction in BMI z-score after 1 and 2 years of intervention	[29]
Lifestyle	Reinehr et al., 2010	Longitudinal study over 5 years following 1-year outpatient lifestyle (dietary, physical activity, and behavioral) intervention in 663 children (mean age 10.6 years, mean BMI z-score 2.46, 55% female)	Younger age (<8 years) predicted greater reduction in BMI z-score over 5 years, while older age (>13 years) predicted the least reduction	[30]
Lifestyle	Hagman et al., 2018	Prospective cohort of 434 youths (mean age 12.4 years, mean BMI z-score 2.4, 64.5% female) who received lifestyle (dietary, physical activity, and behavioral) intervention for 35.9 ± 20.8 months	Male sex and pubertal adolescents predicted poor response (defined as increase in BMI z-score over time), while higher baseline BMI and carriers of FTO allele were protective factors	[31]
Lifestyle	Vourdoumpa et al., 2023	Systematic review of 27 studies involving 7928 children and adolescents with overweight/obesity (age range 4.5–20 years) examining the influence of genetic variants on response to multidisciplinary lifestyle interventions	Single-nucleotide polymorphisms in 24 genetic loci were associated with greater or smaller BMI/body composition changes in response to lifestyle intervention	[32]
Lifestyle	Dubuisson et al., 2012	Retrospective study of 144 children with obesity (mean age 10.5 years, mean BMI z-score 2.73, 59% female) who participated in family-targeted interdisciplinary lifestyle (dietary, physical activity, and behavioral) program who had ≥2 interdisciplinary visits and ≥1 year of treatment	Increased levels of physical activity and daily water intake at baseline predicted greater BMI z-score reduction after 9 months of lifestyle intervention, while higher intake of soft drinks was a negative predictor	[33]
Lifestyle	Southcombe et al., 2023	Systematic review and meta-analysis of 125 studies of dietary intervention in children and adolescents aged 2–18 years with obesity	Dietary interventions with greater energy deficits were associated with greater BMI reductions, while interventions with no specified energy target were associated with slight increase in BMI	[34]
Lifestyle	De Miguel-Etayo et al., 2019	Prospective study of 117 adolescents (mean age 14.62 years, mean BMI z-score 2.61, 56.4% female) following 13-month multidisciplinary lifestyle (dietary, physical activity, and behavioral) intervention	Higher diet quality index scores predicted greater BMI and fat mass index reductions	[35]
Lifestyle	Hart et al., 2010	Prospective study of 72 adolescents (mean age 14.21 years, mean BMI 30.99 kg/m^2^, 73.6% female) following 16-week multidisciplinary lifestyle (dietary, physical activity and behavioral) intervention	Higher initial frequency of intake of vegetables and increased frequency of intake of fruits and reduced-calorie snack foods over the 1st 4 weeks of treatment is associated with greater reduction in BMI	[36]
Lifestyle	Allali et al., 2024	Prospective study of 165 adolescents (mean age 13.3 years, mean BMI z-score 2.32, 61.2% female) following 16-week multidisciplinary lifestyle (dietary, physical activity, and behavioral) intervention	Higher baseline cardiorespiratory fitness predicted greater reductions in weight, BMI, and fat mass, and predicted increase in lean mass following the intervention	[37]
Lifestyle	Tester et al., 2024	Retrospective longitudinal analysis of 733 children and adolescents (mean age 12.1 years, mean BMI z-score 2.2, 51.7% female) with overweight/obesity without diabetes in a weight management clinic including meeting with a provider, dietitian, exercise specialist, and psychologist with baseline HbA1c within 90 days of first visit	Baseline prediabetes predicted greater reduction in BMI percent of the 95th percentile compared to children with normal baseline HbA1c	[38]
Lifestyle	Pinhas-Hamiel et al., 2008	Retrospective study of 134 adolescents with obesity (mean age 13.4 years, 44% female) enrolled in a lifestyle (dietary, physical activity, and behavioral) intervention	Higher baseline fasting insulin, homeostasis model assessment insulin resistance (HOMA-IR) and the presence of obesity or obesity-related comorbidity in both parents were associated with lower likelihood of BMI z-score improvement	[39]
Lifestyle	Uysal et al., 2013	Longitudinal study of 484 children with obesity (median age 11.1 years, mean BMI z-score 2.42, 57% female) who participated in a 1-year lifestyle (dietary, physical activity, and behavioral) intervention	Higher baseline insulin resistance, increased waist circumference and waist-to-height ratio, higher blood pressure, hypertriglyceridemia, and elevated uric acid were negative predictors of BMI z-score reduction	[40]
Lifestyle	Reinehr et al., 2009	Longitudinal study of 248 children with obesity (mean age 10.6 years, mean BMI 2.43, 53% female) who participated in a 1-year lifestyle (dietary, physical activity, and behavioral) intervention	Higher baseline leptin was a negative predictor of reduction in BMI z-score, waist circumference, and percentage body fat following lifestyle intervention	[41]
Lifestyle	Persaud et al., 2023	Longitudinal cohort of 201 children (mean age 9.57 years, mean %BMIp_95_ 113.67, 44.28% female) who participated in a lifestyle-based pediatric weight management intervention (dietary, physical activity, and behavioral)	Household food insecurity was associated with increased BMI and %BMIp_95_ compared to the food-secure group	[42]
Lifestyle	Eliakim et al., 2004	Prospective study of 77 children with obesity (mean age 10.2 years, mean BMI 35.9 kg/m^2^, 49% female) who participated in a 12-month lifestyle (dietary, physical activity, and behavioral) intervention	Higher baseline BMI percentile and parental obesity were associated with less favorable response	[43]
Lifestyle	Heinberg et al., 2010	Prospective study of 104 children and adolescents with obesity (mean age 11.63 years, mean BMI 33.03 kg/m^2^, 65% female) and their caregivers who participated in a 12-week lifestyle (dietary, physical activity, and behavioral) intervention	Lower baseline parental involvement was associated with reduced likelihood of weight loss	[44]
Pharmacotherapy	Bensignor et al., 2023	Post-hoc analysis of adolescents enrolled in the SCALE Teens trial randomized to receive liraglutide (*n* = 125, mean age 14.6 years, mean BMI 35.3 kg/m^2^, 61.9% female) versus placebo (*n* = 126, mean age 14.5 years, mean BMI 35.8 kg/m^2^, 61.9% female) for 56 weeks	Early response to liraglutide (≥4% reduction in BMI at week 16) was a positive predictor for BMI and body weight reduction at week 56 compared to early non-responders	[45]
Pharmacotherapy	Nathan et al., 2015	Post-hoc analysis of pooled data from 2 clinical trials of 32 adolescents (mean age 14.3 years, mean BMI 39.8 kg/m^2^, 69% female) treated with exenatide for 3 months	Female sex and higher baseline appetite were positive predictors of BMI change after 3 months of exenatide treatment	[46]
Pharmacotherapy	Bensignor et al., 2024	Post-hoc analysis of 66 adolescents (mean age 16 years, BMI 36.87 kg/m^2^, 47% female) enrolled in a clinical trial who had achieved ≥5% BMI reduction with meal-replacement therapy and were subsequently randomized to exenatide or placebo for 52 weeks	Lower leptin response to meals at baseline was associated with greater weight loss maintenance in those receiving exenatide	[47]
Metabolic and bariatric surgery	Beck et al., 2025	Prospective, observational cohort study of 73 adolescents (mean age 17.6 years, mean BMI z-score 2.63, female 65.8%) undergoing MBS (87.7% sleeve gastrectomy, 12.3% gastric bypass) followed for up to 30 months post-surgery	Higher preoperative BMI was a negative predictor for achieving a >35% reduction in BMI z-score at 12 months	[48]
Metabolic and bariatric surgery	Mackey et al., 2019	Prospective study of 173 adolescents and young adults (mean age 16.6 years, mean preoperative BMI 50 kg/m^2^, 74% female) undergoing sleeve gastrectomy with self-reported preoperative physical activity levels	Higher preoperative exercise predicted greater weight loss at 6 months and (marginally) 12 months post-surgery; lower preoperative BMI was a positive predictor	[49]
Metabolic and bariatric surgery	Burghard et al., 2024	Retrospective study of 151 adolescents (mean age 15.9 years, 77.5% female) who underwent sleeve gastrectomy	Higher systolic blood pressure predicted greater reduction in absolute BMI and BMI z-score at 6 and 12 months; higher HbA1c predicted greater reduction in BMI z-score at 6 months	[50]
Metabolic and bariatric surgery	Sysko et al., 2012	Prospective study of 101 adolescents with obesity (mean age 15.8 years, mean BMI 47.23 kg/m^2^, 72.3% female) who underwent laparoscopic adjustable gastric banding and were followed for 1 year postoperatively	Higher baseline family conflict was associated with reduced postoperative BMI reduction	[51]

## Data Availability

No new data were created or analyzed in this study.
